# Evaluating the impact of an integrated multidisciplinary head & neck competency-based anatomy & radiology teaching approach in radiation oncology: a prospective cohort study

**DOI:** 10.1186/1472-6920-14-124

**Published:** 2014-06-26

**Authors:** Leah D’Souza, Jasbir Jaswal, Francis Chan, Marjorie Johnson, Keng Yeow Tay, Kevin Fung, David Palma

**Affiliations:** 1 Department of Anatomy & Cell Biology, Schulich School of Medicine & Dentistry, Western University, London, ON, Canada; 2 Division of Radiation Oncology, Department of Oncology, Schulich School of Medicine & Dentistry, Western University, London, ON, Canada; 3 Department of Medical Imaging, London Health Sciences Centre, Victoria Hospital, London, ON, Canada; 4 Department of Otolaryngology - Head and Neck Surgery, London Health Sciences Centre - Victoria Hospital, London, ON, Canada; 5 London Regional Cancer Program, London, ON, Canada

**Keywords:** Anatomy, Education, Radiotherapy, Contouring, Head and neck cancer

## Abstract

**Background:**

Modern radiation oncology demands a thorough understanding of gross and cross-sectional anatomy for diagnostic and therapeutic applications. Complex anatomic sites present challenges for learners and are not well-addressed in traditional postgraduate curricula. A multidisciplinary team (MDT) based head-and-neck gross and radiologic anatomy program for radiation oncology trainees was developed, piloted, and empirically assessed for efficacy and learning outcomes.

**Methods:**

Four site-specific MDT head-and-neck seminars were implemented, each involving a MDT delivering didactic and case-based instruction, supplemented by cadaveric presentations. There was no dedicated contouring instruction. Pre- and post-testing were performed to assess knowledge, and ability to apply knowledge to the clinical setting as defined by accuracy of contouring. Paired analyses of knowledge pretests and posttests were performed by Wilcoxon matched-pair signed-rank test.

**Results:**

Fifteen post-graduate trainees participated. A statistically significant (p < 0.001) mean absolute improvement of 4.6 points (17.03%) was observed between knowledge pretest and posttest scores. Contouring accuracy was analyzed quantitatively by comparing spatial overlap of participants’ pretest and posttest contours with a gold standard through the dice similarity coefficient. A statistically significant improvement in contouring accuracy was observed for 3 out of 20 anatomical structures. Qualitative and quantitative feedback revealed that participants were more confident at contouring and were enthusiastic towards the seminars.

**Conclusions:**

MDT seminars were associated with improved knowledge scores and resident satisfaction; however, increased gross and cross-sectional anatomic knowledge did not translate into improvements in contouring accuracy. Further research should evaluate the impact of hands-on contouring sessions in addition to dedicated instructional sessions to develop competencies.

## Background

In recent decades, a competency-based movement in medical education has emerged as a priority in curriculum development [1]. This movement requires that education is broadened to include defined competency areas with additional emphasis on training and evaluation in practical settings [2].

In the field of radiation oncology, while a competency-based paradigm continues to be gradually integrated into post-graduate curricula, the technology of radiation therapy is evolving at a rapid rate; primarily as a result of the evolution of computer applications and the integration of diagnostic imaging and dose delivery technology [3].

**Figure 1:**
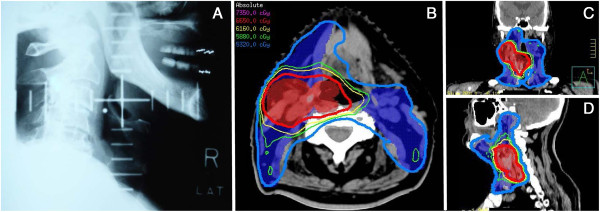
**Evolution of radiotherapy planning.** Historically, radiation treatment planning was based on bony landmarks as seen using 2-dimensional x-ray films **(A)**. Modern radiotherapy allows for sculpting of radiotherapy dose in three dimensions (axial **B**; coronal **C**; sagittal **D**), with differential doses to high-risk (thick red line) and low-risk (thick blue line) areas, and relative sparing of the normal tissues including parotid glands, spinal cord and brain.

The primary goal of radiation therapy is to deliver enough high dose radiation to eradicate the tumor and cancer cells or to palliate symptoms, while avoiding normal tissue injury [3,4]. Prior to the 1990’s, radiation planning was relatively simple, using two-dimensional (2-D) planning based only on x-ray imaging (Figure 1A) [5]. Radiation oncologists who trained in this era relied on radiographs and used bony anatomy to localize the radiotherapy targets. Radiotherapy has progressed from three dimensional conformational radiotherapy (3D-CRT), in which the design and delivery of radiotherapy treatment plans are based on 3-D image data (CT-based) with treatment fields individually shaped to treat only the target tissue, to even more advanced techniques such as Intensity-Modulated Radiotherapy (IMRT, Figure 1B-D) [4,5]. IMRT has the ability to decrease the dose to critical structures, therefore decreasing side effects of radiotherapy, while providing adequate dose to the tumor [5]. However, the success of IMRT is incumbent upon accurate contouring (outlining) of the target volume and organs at risk (OAR) [5]. That is, errors in delineating targets or OAR have significant clinical consequences given the often high doses of radiation and their proximity to healthy normal tissues [5].

Modern radiation oncologists therefore require unique competencies that include 3-D knowledge of normal anatomy and tumor tissue, including its demonstration by diagnostic imaging, and target delineation/treatment planning. Despite technological advancements necessitating advanced 3-D gross and radiologic anatomy knowledge training to enhance competency in radiation treatment planning and delivery, the evolution of many institutions’ radiation oncology postgraduate medical education curricula have not matched this pace.

As is the case for many other post-graduate specialties, radiation oncology residents have not had formal gross anatomy teaching since their first or second year of undergraduate medical education [6]. Furthermore, the hours allotted to anatomy during the undergraduate medical curriculum have decreased steadily over the past several years [6-10]. The literature also indicates that relative to other oncology-related disciplines, radiation oncology is underrepresented in undergraduate medical education, further ill-preparing medical residents entering this discipline [11].

The current model of many radiation oncology training programs operates primarily on an apprenticeship-based model, rather than on formally structured curricula. In general, radiation oncology residents are expected to develop clinical expertise and competency in all aspects of radiotherapy by working one-on-one with a faculty member in the clinic, and attending seminar-based teaching. While the apprenticeship model and experiential-based learning has its merits, its feasibility in ensuring competency development of specific skills such as contouring can be debated. The teaching of contouring skills may require a substantial component of one-on-one teaching time, which may be difficult to schedule amid the competing priorities of regular didactic lectures and clinical care.

To date, limited research has described gross and radiologic anatomy educational interventions within the field of radiation oncology that target development of multiple medical competencies. We conducted this study to determine the efficacy and effectiveness of incorporating an integrated, kinesthetic, multidisciplinary team (MDT)-based head-and-neck educational program for radiation oncology post-graduate trainees, and to assess the effects of the educational program on competency development at multiple levels. The head and neck clinical site was selected as the overarching topic of the educational program given its complex anatomy and geometry, and because the advanced technology of IMRT has been rapidly adopted in the treatment of head-and-neck cancers.

## Methods

### Study design

This study is modeled after a prospective cohort research design. The institutional review board, Western University’s Research Ethics Board (REB), approved this pilot study (REB 102624) under the Delegated Health Sciences Review – Level 2 Category prior to study initiation. The course was developed by members of the Department of Anatomy & Cell Biology, Department of Otolaryngology-Head and Neck Surgery, Department of Radiology, and the Department of Oncology at Western University. The educational intervention involved introducing a formalized integrated, kinesthetic, and MDT-based anatomy and radiology (head-and-neck) curriculum into the postgraduate radiation oncology training at Western University, through the implementation of four seminars addressing head-and-neck anatomic sub-sites that are pertinent to radiation oncologists.

Fifteen postgraduate trainees at the London Regional Cancer Program participated during the 2012-2013 academic year. The study population was inclusive of all on-service postgraduate trainees in the field of radiation oncology; that is, radiation oncology residents of post graduate year (PGY) two through five, and medical physicists. PGY 1 radiation oncology residents were ineligible because these medical residents are off-service from radiation oncology clinical rotations during their first year. Eligible residents were prospectively enrolled with informed consent and letters of information, in accordance with Western University’s REB regulations.

### Study schema

The overall study schema consisted of a baseline evaluation, the teaching intervention, and a follow up evaluation.

The baseline evaluation was conducted prior to the content dissemination during the teaching intervention, and consisted of five components: a contour pre-test, a demographic profile questionnaire, a preferred learning style assessment (measured through Kolb’s Learning-Style Inventory version 3.1), a pre-questionnaire (measured through the use of visual analogue scale (VAS) questions), and finally a short anatomy and radiology boards style knowledge pre-test. Similar components were included for the follow up evaluation: a contour post-test, post-questionnaire (VAS questions and open-ended post-intervention questions), and boards style knowledge post-test.

The teaching intervention was designed to offer a comprehensive overview of head-and-neck anatomy and oncology for complex areas of comprehension, specifically focusing on building participants’ competency of anatomic knowledge and clinical application skill of identifying the anatomic landmarks on cross sectional imaging for target delineation.

During a 4-week period, all participants simultaneously attended three 3-hr seminars on head-and-neck sub-sites: base of skull, oropharynx, and larynx; followed by one 3-hr seminar dedicated solely to the principles of head-and-neck radiation oncology (Table 1). Each of the weekly seminars included a general overview of the gross anatomy and patterns of tumor spread (delivered by the anatomist); CT and MR based diagnostic imaging (delivered by the attending radiologist); radiation oncology nodal levels, commonly observed contouring errors, clinical cases (delivered in tandem by the attending radiologist and radiation oncologist); and principals of radiation oncology (delivered by the attending radiation oncologist). In addition, for the larynx sub-site, an attending head-and-neck surgeon delivered the principles of surgical management in the context of radiation oncology. The simultaneous presence of multiple attending physicians (both as instructors and auditors amongst the audience) facilitated MDT collaboration, contributing to the uniqueness of the educational intervention.

**Table 1:** Teaching intervention

**Topic 1: Base of skull**	**Topic 2: Larynx**	**Topic 3: Oropharynx**	**Topic 4: Principles of radiation oncology**
1. Didactic Gross Anatomy	1. Didactic Gross Anatomy	1. Didactic Gross Anatomy Didactic	1. Organs at risk (OAR)
2. Gross Anatomy Interactive	2. Gross Anatomy Interactive	2. Gross Anatomy Interactive	2. Nodal Levels
3. Didactic Radiology	3. Didactic Radiology	3. Surgical Principles	3. Target Volume Determination
4. Clinical Cases	4. Clinical Cases	4. Clinical Cases	4. Patterns of Spread

Investigators provided participants with the opportunity to observe 3-D anatomic relationships through the direct visualization of human cadavers, so as to better integrate anatomical concepts and assist postgraduate trainees to translate anatomic principles to 2-D diagnostic images with clinical context to radiation oncology priorities and concerns [6]. Participants used anatomic models to manipulate concurrently during the didactic component, and cadaveric specimens were explored in a small-group teaching style around site-specific prosection stations to provide a kinesthetic learning environment.

### Primary outcome measures

To assess the effectiveness of this educational intervention, the following aims and assessment tools were defined:

To assess the educational impact of the teaching intervention on participants’ anatomic and radiographic knowledge, a short anatomy and radiology boards style knowledge test (comprising of 27 multiple choice and identification style questions) was administered immediately prior to and after the seminar series. The boards style knowledge test mimicked the Royal College of Physicians and Surgeons of Canada’s postgraduate training examination with questions of similar difficulty level and format. Each question was time restricted to 30 seconds, and participants were not allowed to revisit previous questions upon proceeding to the next question. Pretest and posttest content was identical, with alterations in the ordering of questions to reduce re-test bias.

To assess the residents’ ability to translate the anatomic and radiographic knowledge potentially gained to the practical skill of contouring, participants were instructed to complete a pre and post contouring test which involved contouring 20 specific anatomical structures on select axial CT images of an anonymized treatment plan. Each structure was selected by investigators to represent an anatomical site fundamental for radiation oncologists to comprehend for target delineation. Contour responses were graded quantitatively with reference to “gold standard” contours defined by a radiation oncologist and radiologist. Pairs of pretests compared to the gold standard versus posttests compared to the gold standard were evaluated using MIMvista software 5.0 (MIM Software, Cleveland, OH, USA). The dice similarity coefficient (DSC) was used to compare the contours to the gold standard. The DSC measures the spatial overlap between two segmentations, A and B target regions, and is defined as DSC (A,B) = 2(A∩B)/(A + B) where ∩ is the intersection [12].

Pre-test and post-test questionnaires assessed resident satisfaction of incorporating the teaching intervention into the residency educational curriculum. Both questionnaires included mirrored questions using the VAS assessment tool to observe a change in self-reported participant scores of self-confidence/comfort level with competencies in target delineation, head-and-neck anatomy knowledge, and reading radiographs for each selected topical sub-site. The post-questionnaire included additional unique VAS questions and qualitative (open-ended) questions for formative assessment of the teaching intervention. A VAS is an assessment tool (with a minimum score of 0 to a maximum score of 10) that measures a characteristic or attitude that is believed to range across a continuum of values that cannot easily be directly measured [13,14]. The VAS was selected as the measurement instrument, as opposed to the Likert Scale, because participants' responses did not have to be categorized or were not qualified in discrete terms [14].

### Statistical analysis

All paired data were compared using the Wilcoxon Signed-Rank test. The non-parametric technique was considered because it makes inferences about entire populations rather than population parameters, and makes fewer assumptions about the nature of the data (equal variance, normal distribution). To assess whether a distribution in change of scores was attributed to the independent variables (i.e. gender, profession, previous head-and-neck clinical rotation, and learning style), further analyses was performed. The Wilcoxon Rank Sum test was then used to assess whether there was a distribution of change in scores for the following variables: gender and previous head-and-neck clinical rotation. A head-and-neck clinical rotation can be defined as a dedicated radiation oncology clinical service providing care and treatment planning for head and neck cancer patients. This statistical analysis was selected because it tests the hypotheses about the difference between two independent/un-paired population means using the difference between two sample means and does not require that populations have normal distributions. The Kruskal-Wallis test was utilized to assess whether a distribution of change in scores was attributed to the variables profession and learning style, because of comparison between three or more independent sub-groups.

Quantitative data without a comparative value (i.e. posttest only) was reported with the median score and interquartile range. Using participants’ post-test open-ended responses, themes were identified to establish strengths and areas of improvement. All statistical analyses were performed using SAS 9.2 (Cary, North Carolina, USA) with two-sided statistical testing at the 0.05 significance level.

## Results

**Figure 2:**
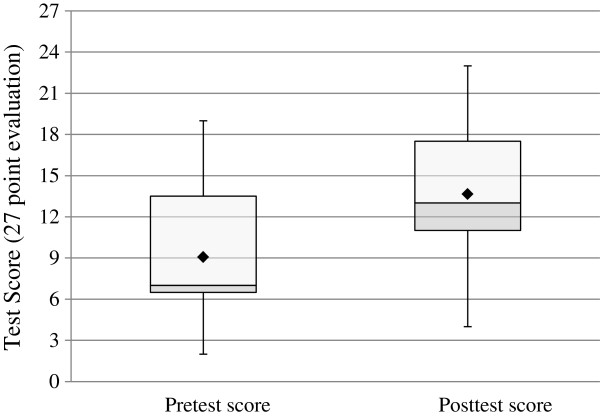
**Mean and median pretest vs. posttest anatomy and radiology boards style knowledge evaluation scores.** The box outlines the upper and lower quartiles, the horizontal line inside the box is the median, the upper and lower whiskers respectively represent the maximum and minimum values, and the diamond symbol represents the mean. The median pretest score was 7 (IQR 6.5-13.5) vs. a posttest median of 13 (IQR 11-17.5), p < 0.0001 for difference by paired analysis.

All potential eligible participants participated in the study (n = 15). The characteristics of the study participants are shown in Table 2. Of the 11 participants who had been previously exposed to a gross anatomy course or gross anatomy lab, all reported the type of exposure as: lecture/didactic anatomy based instruction, cadaveric dissection, and cadaveric prosected specimens.The median anatomy and radiology boards style knowledge pretest score was 7 (IQR 6.5-13.5) vs. a posttest median score of 13 (IQR 11-17.5), p < 0.0001 (Figure 2). There was no significant difference observed in change in scores when comparing gender, profession, learning style, or participants with vs. without previous head-and-neck clinical rotation exposure.

**Table 2:** Participant characteristics (n = 15)

**Variable**	**Participants (%)**
Male	7 (47)
Female	8 (53)
Medical physicist	3 (20)
Junior PGY radiation oncology resident (PGY 2-3)	6 (40)
Senior PGY radiation oncology resident (PGY 4-5)	6 (40)
Previous head-and-neck clinical rotation	9 (60)
Previous gross anatomy course	11 (73)
Previous gross anatomy lab	11 (73)
Preferred learning style: converging	6 (40)
Preferred learning style: diverging	1 (7)
Preferred learning style: assimilating	7 (47)
Preferred learning style: accommodating	1 (7)

Results of the contouring assessments are shown in Table 3. A statistically significant improvement in contouring accuracy was observed for only 3 of the 20 structures, whereas no statistically significant improvement in contouring was evident for the majority of structures.

**Table 3:** Comparison of pre-post contouring accuracy results (through dice similarity coefficient)

**Structure**	**N**	**Dice similarity coefficient: pre-test median**	**Dice similarity coefficient: post-test median**	**P-value**
Clivus	15	0.00	0.46	0.020
Hyoid bone	15	0.71	0.76	0.241
Optic chiasm	15	0.00	0.00	0.219
Sphenoid sinus	15	0.84	0.83	0.492
Thyroid gland	15	0.61	0.65	0.879
Left cochlea	15	0.00	0.00	0.578
Left jugular foramen	15	0.22	0.62	0.577
Left lateral pterygoid muscle	15	0.00	0.03	0.160
Left lens	15	0.67	0.77	0.026
Left level v lymph nodes	15	0.43	0.52	0.519
Left piriform sinus	15	0.13	0.57	0.005
Left submandibular gland	15	0.80	0.81	0.303
Right arytenoid cartilage	15	0.00	0.00	0.547
Right common carotid artery	15	0.05	0.29	0.865
Right level iIa lymph nodes	15	0.28	0.13	0.376
Right masseter muscle	15	0.81	0.83	0.720
Right optic nerve	15	0.58	0.60	0.330
Right parotid gland	15	0.66	0.69	0.985
Right tonsillar fossa	15	0.07	0.04	0.588
Right vocal cord	15	0.00	0.10	0.250

**Figure 3:**
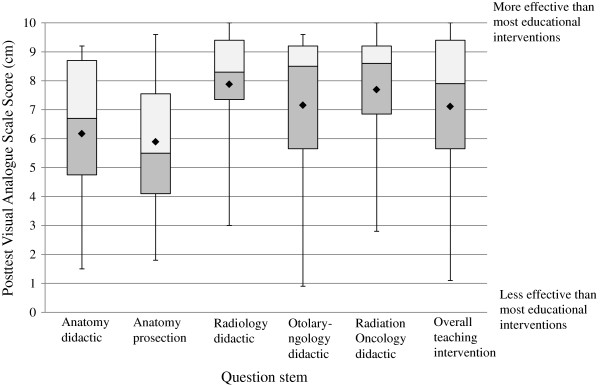
**Posttest questionnaire results of the educational effectiveness of the multidisciplinary components and overall teaching intervention.** Results of the educational effectiveness of the distinct multidisciplinary components and the overall teaching intervention as gathered from the posttest only visual analogue scale (VAS) questionnaire. The box outlines the upper and lower quartiles, the horizontal line inside the box is the median, the upper and lower whiskers respectively represent the maximum and minimum values, and the diamond symbol represents the mean.

Significant improvement in the mean change of VAS scores was observed for self-reported confidence/comfort level in each of the following competencies: tumor/target delineation, anatomic knowledge of the sub-sites, and reading radiographs in the anatomic sub-sites. A description of the questions along with the reported median and associated statistical significance is found in Table 4.Figure 3 graphically represents the scores from select post-intervention only VAS questions. A general trend of high resident satisfaction was observed. While the educational effectiveness of all components were rated above the median of the assessment tool (indicated by the 5 cm point on the VAS scale), the educational effectiveness of the anatomy didactic and prosection components rated comparatively lower than the other components. The educational effectiveness of the anatomy didactic and prosection components had a median score of 6.70 (IQR 1.50-9.20) and 5.50 (IQR 1.80-9.60) respectively. The median score for the educational effectiveness of the radiology didactic component was 8.30 (IQR 3.00-10.00). The educational effectiveness of the otolaryngology didactic component median score was 8.50 (IQR 0.90-9.60). A median score of 8.60 (IQR 2.80-10.00) was observed for the educational effectiveness of the radiation oncology didactic component.

**Table 4:** Comparison of pre-post Visual Analogue Scale (VAS) questions

**Q#**	**Description**	**Pre-VAS median**	**Post-VAS median**	**Wilcoxon S-statistic**	P-value*
1a	Rate your self-confidence/comfort level with target/tumor delineation	4.80	6.70	44.5	0.0087
2a	Rate your self-confidence/comfort with head and neck anatomy for the base of skull anatomical region	1.90	5.70	55.5	0.0005
2b	Rate your self-confidence/comfort with head and neck anatomy for the oropharynx anatomical region	2.80	6.60	52.5	0.0001
2c	Rate your self-confidence/comfort with head and neck anatomy for the larynx anatomical region	2.70	6.40	41.0	0.0068
3a	Rate your self-confidence/comfort with reading radiographs (CT, MRI) for the base of skull anatomical region	2.00	5.30	52.0	0.0015
3b	Rate your self-confidence/comfort with reading radiographs (CT, MRI) for the oropharynx anatomical region	3.90	5.90	49.5	0.0027
3c	Rate your self-confidence/comfort with reading radiographs (CT, MRI) for the larynx anatomical region	2.90	5.50	53.0	0.0012

Overall, the educational effectiveness of the teaching intervention as a whole, was rated positively with a median score of 7.90 (IQR 1.10-10.00) when compared to other educational seminars offered by Western University’s Radiation Oncology postgraduate program. The educational seminars used as a comparison occurred during academic half days, in which trainees usually attend didactic lectures/case studies, followed by a question/answer period. These comparative educational seminars are not MDT based or kinesthetic.

Qualitative (open-ended) results from the posttest questionnaire provided formative evaluation and identified areas of improvement from the participants’ perspectives. When asked “how, if at all, did the teaching intervention alter or inform your practice?” participants’ responses could be grouped into the following themes: feeling more capable of identifying anatomic structures and possible routes of spread, having a better understanding of contouring and nodal levels, and having more confidence with planning and treating. Suggestions to improve the teaching intervention included more teaching time for the radiology didactic component and the anatomy sessions, and the integration of contouring practice.

## Discussion

Anatomic expertise is an essential foundational competency in radiation oncology, specifically for appropriate workup, staging, and treatment. Radiation oncologists are required to understand the relationship between target tissues, OAR, and how the surrounding anatomy dictates patterns of local disease spread when designing radiation therapy treatment plans [6,15]. Results of this study demonstrate an integrated, kinesthetic, MDT-based program enriches the traditional radiation oncology postgraduate curriculum, and provides the ability to assess learner performance across multiple competencies. Trainees’ knowledge and understanding of anatomy and radiology improved after being exposed to the intervention, thereby developing the key competency of establishing and maintaining clinical knowledge appropriate to practice. However, we observed suboptimal development of contouring competency, as indicated by the poor ability to apply core medical knowledge to the practical skill of accurate contouring.

Key technological innovations, such as the introduction of inverse treatment planning systems with IMRT have modified the daily practice of radiotherapy. Such advancements in technology demand stronger competencies such as contouring accuracy and target delineation. Gregoire et al. note that target volume selection and delineation undoubtedly represent the most dramatic change in clinical approach, as compared with the former 2D approach [16]. Furthermore, with evidence demonstrating variation in target delineation between expert clinicians, there is a need for specialized training that includes competencies such as anatomical knowledge and target delineation as a requirement of the radiation oncology postgraduate medical education curriculum [17-20].

A survey of resident education in intensity modulated radiation therapy (IMRT) described by Malik *et al*. revealed that most (70%) respondents (from among 77 accredited radiation oncology residency programs) desired an increase in IMRT didactics, while 1/5 of the 86.9% respondents with hands-on IMRT experience lacked formal training in IMRT. There have since been several studies investigating educational techniques to improve target delineation among residents [21]. Institutions such as Duke University Medical Center have independently begun to address the need to formally integrate competency training and measure specific educational outcomes during the postgraduate clinical years [6]. A study from Memorial Sloan-Kettering demonstrated improved head-and-neck target delineation skills among 11 participants after didactic seminar series and hands-on practical image segmentation sessions [17]. Another study of 12 staff radiation oncologists demonstrated that an interactive, hands-on training session improved consistency in target delineation for cervical esophageal cancer [22].

While our contouring results were suboptimal and not consistent with those of previous studies, this can primarily be attributed to the lack of integrated contouring teaching during seminars. The inability of participants to translate the knowledge to the competency of contouring highlights a misalignment of the teaching interventions curriculum objectives and evaluation methods. That is, despite providing an integrated, kinesthetic, MDT learning environment, no formal contouring instruction or practice was integrated into the seminar series. This shortcoming can be attributed to the restricted time frame allotted for seminars. Although significant improvement in contouring accuracy was only observed for 3 out of 20 anatomic structures, it is important to compare how factors such as density, material, and/or size could have impact on the level of difficulty for the anatomic structures. For example, (both pre and post) contouring accuracy was generally more accurate for bone and larger spaces/sinuses, while soft tissues (e.g. muscle or mucosa) generally demonstrated less accurate contouring. The results may furthermore be reflective of the evaluation parameters. That is, participants were not supervised during the contouring evaluation to ensure all questions were attempted. It was not determined whether DSC pre and/or post-test median scores of 0.000 were reflective of participants skipping the question, or as a result of not having the anatomy/radiology knowledge. The contouring results may also be reflective of the varying effort levels among participants; given that participants had the flexibility to complete this evaluative component during a two week time frame (pre and post contour evaluations were respectively conducted up to two weeks prior to and after the teaching intervention). Time of completion of the evaluation (e.g. post call versus during a designated research time) may have also influenced outcomes. Despite these shortcomings, quantitative VAS results revealed that participants felt more confident at contouring and treatment planning, and had a better understanding of anatomic structures and nodal levels.

These results should remind educators that educational interventions that incorporate sequenced didactic and kinesthetic components are more effective in developing procedural competencies than didactic sessions alone. As well, these findings should remind curriculum developers that targeted efforts towards developing each competency should be implemented. That is, it is flawed to assume that by establishing and maintaining clinical knowledge and attitudes appropriate to practice, trainees will also demonstrate proficient and appropriate use of practical skills (diagnostic and/or therapeutic). The local implication of the results and the supported literature should also prompt postgraduate radiation oncology programs with an apprenticeship-based model to increase the number of formalized hours dedicated to teaching core skills such as: anatomy, radiology, *and* contouring; as the former two components are alone not sufficient to improve the latter. On the basis of these findings, we plan to incorporate hands-on contouring practice in future teaching interventions.

While the small-group anatomy stations constituted the kinesthetic component of the educational intervention, and the literature acknowledges the importance of anatomy in radiation oncology, post-questionnaire VAS results indicated lower educational effectiveness scores of the anatomy didactic and prosection components as compared to other components. Investigators conducted follow up exit interviews and examined qualitative feedback to elucidate this finding. Participants expressed time constraints in the didactic component, and difficult emotions associated with cadaveric specimens which revealed identity (e.g. a face) as attributing potential factors.

The limitations of this study include confounders common to all pretest and posttest designs, including maturation effects (i.e. self-directed learners read about the subject prior to/after the seminar series), and repeated measurement effect (i.e. the pretest affects the posttest results). Moreover, the single-institution (lack of control group) small sample size can be considered a limitation (as sufficient power for statistical analysis becomes of concern), but the sample size is comparable to other radiation oncology educational interventions in the literature. Study investigators recognize the potential shortcoming of the quantitative evaluation tools that were used, which had not been previously tested for validity or reliability. Furthermore, we did not assess longer term outcomes to determine whether the teaching intervention was associated with retention. Postgraduate medical education researchers will also agree that commitment from participants is often difficult to obtain, as reflected by the small number of reports of resident educational interventions with evaluation of retention [15]. Finally, the very nature of educational interventions involves multiple simultaneous sequential learning methods and experiences. As such, it may prove difficult to elucidate which components of the intervention (e.g. instructional methods vs. experiences) helped to demonstrate a specific outcome. These shortcomings have been addressed and participant feedback incorporated as the initial pilot study has become regularly incorporated into the 2012-2013 oncology academic half day schedule, and into the design of a national level workshop.

Study limitations notwithstanding, most participants found that the teaching intervention was a valuable experience. The positive outcomes as well as the inadequacies of this study may inspire curriculum designers, educators, program directors, and students themselves, to consider educational models that advance beyond apprenticeship-focused learning, toward multidisciplinary formal designs with transparency of the required competencies. The integration of similar educational interventions into current apprenticeship-based programs is achievable with majority support from the department (program directors, attending physicians, and trainees), interdepartmental collaboration (e.g. radiology), and re-organization of traditional academic half-days. The results and implications of this study, coupled with the identified need for change at the postgraduate education level as identified through the literature, proposes a revision and harmonization of the core curricula for radiation oncologists, to reflect the rapid development of radiotherapy technology, and be inclusive of anatomy, radiology, and contouring instruction. Based on the results reported herein, the course has been modified and developed into a Canadian national “Anatomy and Radiology Contouring Bootcamp”, with changes including: increased length of anatomy prosection interaction, dedicated hands-on-contouring sessions with real-time feedback, and improved integration of teaching across MDT specialties. Future work will evaluate the improvements in anatomy and contouring knowledge for participants attending the bootcamp.

## Conclusion

Incorporating a kinesthetic MDT anatomy and radiology educational intervention, which targets multiple competencies, into an existing apprenticeship-based radiation oncology postgraduate curriculum has the ability to identify competencies and areas requiring further attention. The results of our study represent short-term core medical knowledge improvements with minimal procedural improvement in target delineation skills. Overall, results indicated positive satisfaction amongst participants. Further evaluative research is required to incorporate content and experiential-based learning refinement, longer term follow-up, and multi-institutional collaboration, so as to foster competency development of residents at all levels, with the goal of improving radiotherapy precision and ultimately patient outcomes.

## Abbreviations

MDT: Multidisciplinary team; IMRT: Intensity modulated radiotherapy; ENT: Otolaryngology; DSC: Dice similarity coefficient; VAS: Visual analogue scale; 3D - CRT: Three dimensional conformational radiotherapy; 2-D: Two dimensional; OAR: Organs at risk; REB: Research ethics board; CT: Computed tomography; MR: Magnetic resonance imaging; IQR: Interquartile range.

## Competing interests

The author(s) declare that they have no (financial or non-financial) competing interests.

## Authors’ contributions

LD provided substantial contribution to the project design, implementation, and evaluation. LD also had a main role in writing this manuscript. JJ, chief resident also assisted in the project development and implementation phases. JJ was involved in editing this manuscript. FC assisted in the project design and implementation. FC was one of the primary anatomists who delivered content material during the study. MJ assisted with the project design, implementation, and manuscript drafting. MJ also served as a primary anatomist who delivered content material during the study. KYT assisted with project implementation, serving as the primary radiologist who delivered content during the study. KYT has also been involved with the manuscript drafting process. KF assisted with project design and implementation. KF served as the primary surgical oncologist who delivered content during this study. KF was also involved with the manuscript draft. DP, research supervisor, assisted with the project design, implementation, and evaluation. DP also served as the primary radiation oncologist who delivered content during this study. All authors have read and approved the final manuscript.

## Authors’ information

LEAH D'SOUZA is a graduate from the Department of Anatomy and Cell Biology, Clinical Anatomy Division at the Schulich School of Medicine and Dentistry at Western University in London, ON, Canada.

JASBIR K. JASWAL is a Radiation Oncology (chief, PGY-5) resident at the London Regional Cancer Program, Radiation Oncology Division of Oncology, Schulich School of Medicine and Dentistry, Western University, London, ON, Canada. She is also currently enrolled in the Masters of Medical Education Leadership program through the University of New England, Maine.

FRANCIS CHAN (1950-2013*) was an assistant professor in the Department of Anatomy and Cell Biology at the Schulich School of Medicine and Dentistry at Western University in London, ON, Canada.

MARJORIE JOHNSON is an assistant professor in the Department of Anatomy and Cell Biology, Associate Chair of the Division of Clinical Anatomy and Director of the Clinical Anatomy graduate program at the Schulich School of Medicine and Dentistry at Western University in London, ON, Canada.

KENGYEOW TAY is an assistant professor in the Department of Medical Imaging, Radiology, at the Schulich School of Medicine & Dentistry at Western University, and a radiologist in the Department of Medical Imaging, Radiology, London Health Sciences Centre, London, ON, Canada

KEVIN FUNG is an associate professor in the Department of Otolaryngology –Head & Neck Surgery at the Schulich School of Medicine and Dentistry at Western University, and the site chief in the Department of Otolaryngology - Head & Neck Surgery - Victoria Hospital, London Health Sciences Center, London, ON, Canada

DAVID PALMA is a radiation oncologist at the London Regional Cancer Program and clinician-scientist at the Ontario Institute for Cancer Research. He has an academic focus on precision radiotherapy.

## Pre-publication history

The pre-publication history for this paper can be accessed here:

http://www.biomedcentral.com/1472-6920/14/124/prepub
